# Reduced *Clostridioides difficile* infection in a pragmatic stepped-wedge initiative using admission surveillance to detect colonization

**DOI:** 10.1371/journal.pone.0230475

**Published:** 2020-03-19

**Authors:** Lance R. Peterson, Sean O’Grady, Mary Keegan, Adrienne Fisher, Shane Zelencik, Bridget Kufner, Mona Shah, Rachel Lim, Donna Schora, Sanchita Das, Kamaljit Singh

**Affiliations:** 1 Department of Medicine, Division of Infectious Diseases, NorthShore University HealthSystem, Evanston, Illinois, United States of America; 2 Department of Pathology and Laboratory Medicine, Division of Microbiology, NorthShore University HealthSystem, Evanston, Illinois, United States of America; 3 Department of Infection Control, NorthShore University HealthSystem, Evanston, Illinois, United States of America; 4 Chief Clinical Operations Officer, NorthShore University HealthSystem, Evanston, Illinois, United States of America; 5 Department of Nursing, NorthShore University HealthSystem, Evanston, Illinois, United States of America; Azienda Ospedaliera Universitaria di Perugia, ITALY

## Abstract

**Background:**

*Clostridioides difficile* Infection (CDI) is a persistent healthcare issue. In the US, CDI is the most common infectious cause of hospital-onset (HO) diarrhea.

**Objective:**

Assess the impact of admission testing for toxigenic *C*. *difficile* colonization on the incidence of HO-CDI.

**Design:**

Pragmatic stepped-wedge Infection Control initiative.

**Setting:**

NorthShore University HealthSystem is a four-hospital system near Chicago, IL.

**Patients:**

All patients admitted to the four hospitals during the initiative.

**Interventions:**

From September 2017 through August 2018 we conducted a quality improvement program where admitted patients had a peri-rectal swab tested for toxigenic *C*. *difficile*. All colonized patients were placed into contact precautions.

**Measurements:**

We tested admissions who: *i*) had been hospitalized within two months, *ii*) had a past *C*. *difficile* positive test, and/or *iii*) were in a long-term care facility within six months. We measured compliance with all other practices to reduce the incidence of HO-CDI.

**Results:**

30% of admissions were tested and 8.3% were positive. In the year prior to the initiative (Period 1) there were 63,057 admitted patients when HO-CDI incidence was 5.96 cases/10,000 patient days. During the 12-month initiative (Period 2) there were 62,760 admissions and the HO-CDI incidence was 4.23 cases/10,000 patient days (*p* = 0.02). There were no other practice or antibiotic use changes. Continuing admission surveillance provided a HO-CDI incidence of 2.9 cases/10,000 patient days during the final 9 months of 2018 (*p*<0.0001 compared to Period 1), equaling <1 case/1,000 admissions.

**Limitations:**

This was not a randomized controlled trial, and multiple prevention practices were in place at the time of the admission surveillance initiative.

**Conclusion:**

Admission *C*. *difficile* surveillance testing is an important tool for preventing hospital-onset *C*. *difficile* infection.

**Registration:**

This quality improvement initiative is registered at ClinicalTrials.gov. The unique registration identifier number is NCT04014608.

## Introduction

*Clostridioides* (formerly *Clostridium*) *difficile* is an important health care-associated pathogen and the agent of *C*. *difficile* infection (CDI), having a spectrum of disease ranging from moderate or severe diarrhea to pseudomembranous colitis, toxic megacolon, and death [1]. The burden of CDI has not decreased in the United States or worldwide [2–4]. CDI has surpassed methicillin-resistant *Staphylococcus aureus* (MRSA) as the most common cause of health care-associated infection [3,4]. CDI has significant morbidity and mortality [5], with the median risk of death after infection being 19% (range, 8% to 53%). Survivors of CDI also experience significant change in their behavioral lifestyles [6]. A current CDI prevention hypothesis is that active surveillance testing (AST) for *C*. *difficile* colonization at the time of admission may lower hospital onset (HO) disease rates [7], with a single report testing this hospital-wide intervention [8].

MRSA and *C*. *difficile* have similar epidemiologic characteristics since both microorganisms contaminate the hospital environment [9,10], asymptomatically colonized patients are a significant source of microbial spread to others [9, 11–13], and colonization by these organisms is the first step toward clinical infection [14–17]. Since large studies have demonstrated that AST at admission can lower MRSA clinical disease rates [18], it would be expected that *C*. *difficile* could respond to similar practice, with resulting lower CDI rates [19]. We had previously demonstrated the benefit of admission MRSA AST on lowering clinical infection [20,21], and our hypothesis was that this approach also would be successful for CDI. Thus, the goal of our pragmatic, stepped-wedge infection control initiative was to determine if admission AST for toxigenic *C*. *difficile* would lead to a reduced hospital onset CDI (HO-CDI) incidence at our healthcare organization. After identification of *C*. *difficile* carriers, we postulated that placing them into contact precautions would lead to significantly lower CDI rates and improved patient safety. A secondary goal was to assess the impact of admission testing for *C*. *difficile* colonization in a setting where many other practices (e.g., bleach cleaning of rooms, use of required soap/water hand hygiene for CDI patients, hand hygiene monitoring, portable ultraviolet (UV) light room disinfection, and monitoring of room cleaning) were already in place. We sought to determine the practice/intervention(s) that may have the most impact. Another secondary goal was to demonstrate that adding AST to the Infection Control program for HO-CDI could reduce disease burden to very low levels even when a nucleic acid amplification test (NAAT) was the only laboratory assay used for diagnosis of *C*. *difficile* infection. The investigators were successful in achieving their goals.

## Methods

### Quality improvement initiative approach

NorthShore University HealthSystem (NorthShore) is a four-hospital system with 789 inpatient beds located north of Chicago, IL. From September 2017 through August 2018 we conducted a quality improvement program where admitted patients had a peri-rectal swab collected for toxigenic *C*. *difficile* (TCD) and tested using the cobas^®^ Cdiff Test (Roche Molecular Diagnostics, Pleasanton, CA), a NAAT that detects toxin B gene (*tcd*B). The test and its use for peri-rectal swabs previously was validated for admission testing [22]. All patients who tested positive were placed into isolation (contact) precautions until discharge. No specific action was taken until admission test results were reported.

Our quality improvement Infection Control initiative was a pragmatic stepped-wedge program designed to reduce the rate of CDI in hospitalized patients. The baseline 12 months (Period 1) followed the established practices described in Table 1, which had been in place since April 2014. These are based on Centers for Disease Control and Prevention (CDC), Infectious Disease Society of America (IDSA), and Society for Healthcare Epidemiology of America (SHEA) guidance [23,24]. From July 2016 through December 2016 a pilot initiative was performed at one of the four NorthShore hospitals to evaluate the potential added benefit of AST at admission for toxigenic *C*. *difficile* (Patel PA, Singh R, Vernon M, Schora D, Wang C, Doganay B, et al. Prevalence and risk factors for asymptomatic colonization with *Clostridium difficile* among hospitalized patients. In Program and Abstracts, Microbe 2018. The American Society for Microbiology, June 7–11, 2018. Atlanta, GA. Poster presentation 701}. Based on these results, a risk-based algorithm was developed whereby patients at all 4 hospitals were tested if they had been previously hospitalized within two months, and/or had a past *C*. *difficile* positive test, and/or were in a long-term care facility in the prior six months. The algorithm had a sensitivity of 78.1% (95% confidence interval, CI = 70.3%– 84.3%) for detecting patients colonized with toxigenic *C*. *difficile*, compared to testing all admissions. It was built into our electronic health record (EHR; Epic Systems, Verona, WI) as an automated nursing best practice alert that signaled each time a patient with the appropriate risk profile was admitted to the hospital, as we had done for our MRSA AST [25]. Perirectal samples were taken at the time of admission by nursing personnel. Testing was performed six days per week (excluding Sundays) with results reported in less than 24 hours from the time of admission. A positive test was reported with the following comment: *Positive*: *C*. *difficile toxin gene detected by PCR*. *Test is positive for C*. *difficile carriage*. *Only treat for CDI if patient has significant diarrhea*.

**Table 1:** Infection control practices followed in baseline and intervention initiative periods.

Infection Prevention Practice	Technique Used	Target of Practice	Monitoring
Terminal Bleach Cleaning	10% Sodium Hypochlorite (bleach; v/v) used to wipe all flat surfaces to ceiling after routine terminal room cleaning	All rooms with *C*. *difficile* test positive patients and other MDRO pathogens	Ongoing monitoring of compliance reviewed monthly by Infection Control Professional staff
Soap and Water Hand Hygiene	Dedicated room signage indicating all hand hygiene was to be done with soap and water	All rooms with *C*. *difficile* test positive patients	Ongoing monitoring of compliance by Infection Control Professional staff
Hand Hygiene Compliance	Direct observation by Infection Control Professional and Nursing staff	Hospital wide	Monitoring of hand hygiene performance at room entry and exit reviewed monthly
Personal Protective Equipment (PPE) Compliance	Direct observation by Infection Control Professional and Nursing staff	Hospital wide	Continuous monitoring of PPE use at room entry reviewed monthly
Portable UV Room Disinfection	Tru-D portable UV units ((Tru-D Smart UVC, Memphis, TN) used at sporicidal setting	Placed in room of *C*. *difficile* test positive patient after discharge as well as other rooms on high-rate CDI patient units	Monthly comparison of patient room locations and corresponding use of portable UV Tru-D units
Room Cleaning Compliance	Ultraviolet mark spot test removal following terminal room cleaning	Hospital wide: 10 of 40 high touch room sites tested for each monitoring	Compliance requires 80% of pre-cleaning spots removed, or room is re-cleaned
Physician contact for inappropriate test ordering	Infection Control Professional contacts ordering physician	Contact when no appropriate indication for CDI testing*	Reviewed monthly by Infection Control Professional staff
Targeted Admission *C*. *difficile* Surveillance†	Peri-rectal swab collected at admission and tested using real-time PCR for presence of toxigenic *C*. *difficile*	Patients who were hospitalized within two months, had a past *C*. *difficile* positive test, and/or were in a long-term care facility within the prior six months	Monthly comparison of patients identified as needed admission testing with those having a sample collected and processed

* Appropriate testing was defined as done on patients with ≥3 diarrheal stools in 24 hours, plus no other reason for diarrhea, plus abdominal pain or fever or elevated leucocytes, plus recent antibiotics or hospitalization or past history of CDI or nursing home stay.

† Admission surveillance testing only performed during year two of this report. All other practices were performed in both period one and period two.

### Time periods for analysis

Monitoring of the impact of this initiative in comparison to other Infection Control practices targeted toward CDI was done for the 12 months following implementation at all 4 hospitals; the same monitoring for all other practices was done during the baseline period. The two comparison time periods for this initiative were the baseline from August 2016 through July 2017 (Period 1), and the intervention initiative from September 2017 through August 2018 (Period 2). August 2017 was considered a transition month when hospital personnel were educated and the new practice deployed, and not included in the comparisons. Following Periods 1 and 2 we continued the same monitoring of admission testing on the incidence of HO-CDI for an additional four months, through the end of December 2018. During the entire period of time the clinical microbiology laboratory used only the Xpert^®^ *C*. *difficile* NAAT (Cepheid, Sunnyvale, CA) for the diagnosis of clinical infection.

### Data recording

The outcome of this AST intervention was based on the rate of HO-CDI and reported as cases/10,000 patient days. A HO-CDI case was defined as any patient with a diarrheal stool specimen submitted for testing ≥3 days after admission that tested positive by the Xpert^®^ *C*. *difficile* test, including those testing positive at the time of admission. A patient day was counted as any day when a bed in the hospital was occupied, excluding beds in the nursery, pediatrics, same day surgery, psychiatry, and the emergency department. Other ongoing practices that had potential for significant positive impact on HO-CDI (Table 1) were monitored for percentage compliance with the recommended practice and results were compared between Periods 1 and 2. Antimicrobial agent use was monitored across all four hospitals and recorded as days of therapy (DOT)/1,000 patient days. We compared the rate of testing for clinical CDI between Periods 1 and 2 with the rate of diagnosed disease as well as the rate of negative tests in these two periods in order to evaluate if diagnostic testing may have been responsible for the observed disease reduction.

### Statistical considerations

Statistical analyses were performed with the use of SAS 9.4. HO-CDI was the dependent variable [26]. Categorical variables were assessed using Chi-squared testing (Fisher’s exact testing for counts less than 5). We used a Cochran-Armitage Test for trend analysis that displays a *p*-value [27]. A *p*≤0.05 was used to determine significance. The Pearson correlation coefficient is used to measure the strength of any practice association with reduction of HO-CDI.

### Human ethical consideration

This Infection Control quality improvement initiative was considered exempt from full Institutional Review Board consideration because *i*) the purpose was to produce a new strategy or intervention, *ii*) it was conducted by clinicians and staff who provide care and are responsible for the performance of quality improvement, *iii*) it was designed with the intent to implement improvement for the hospital, *iv*) it involved the population ordinarily seen in the work setting where the project took place, *v*) the planned activity only required consent that is normally sought in clinical practice and the activity could be considered part of usual care, *vi*) the patients where the planned activity took place could potentially benefit from the project, and *vii*) the burden of participating in the activity can be considered acceptable or ordinarily expected when reforms are being introduced to the way care is provided [28]. The hospital’s ethics committee approved infection control initiatives to improve the prevention of *C*. *difficile* infection as a general quality improvement initiative in February, 2009.

### Registration

This quality improvement initiative is registered at ClinicalTrials.gov. The unique registration identifier number is NCT04014608. Since this was considered a quality improvement initiative and not a research program the study was not registered until a decision was made to undertake a formal analysis and publish the results, which occurred after the period of time described in this report. The authors confirm that all ongoing and related trials for this intervention are registered. Hospitalized patients covered in this report are those who were inpatients between October 1, 2015 and December 31, 2018.

Patients were all provided with a written document indicating the infection control practice being performed, and all those sampled provided verbal consent prior to swabbing. The nursery and pediatric nursing units were not part of this initiative.

### Role of the funding source

There was no external funding for this program and all work was done as part of the duties expected from NorthShore employees. The initiative was designed by Infection Control.

## Results

**Fig 1:**
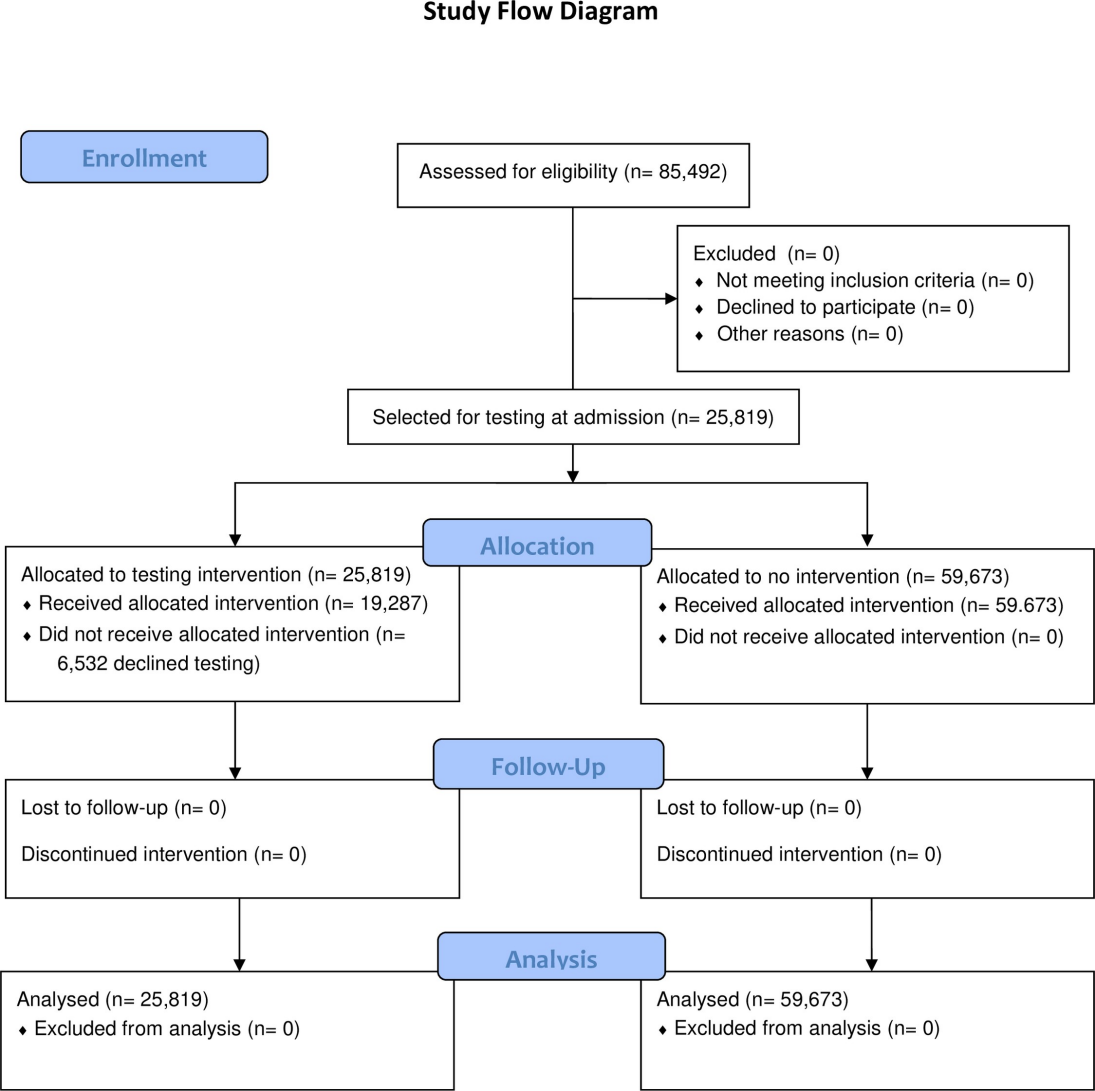
Participant flow diagram for the intervention portion of this report.

In the pilot investigation, 2,024 admissions were tested and the incidence of HO-CDI decreased from 14.64/10,000 patient days in the 1-year prior to the pilot to 6.67/10,000 patient days (54.4% reduction; *p* = 0.017) during the 6-months of universal admission testing (Patel PA, Singh R, Vernon M, Schora D, Wang C, Doganay B, et al. Prevalence and risk factors for asymptomatic colonization with *Clostridium difficile* among hospitalized patients. In Program and Abstracts, Microbe 2018. The American Society for Microbiology, June 7–11, 2018. Atlanta, GA. Poster presentation 701). This provided the needed patient risk data for developing a targeted admission testing algorithm as well as confidence in moving forward to deploy this initiative at all four NorthShore hospitals. A participant flow diagram for this project is in Fig 1.

**Fig 2:**
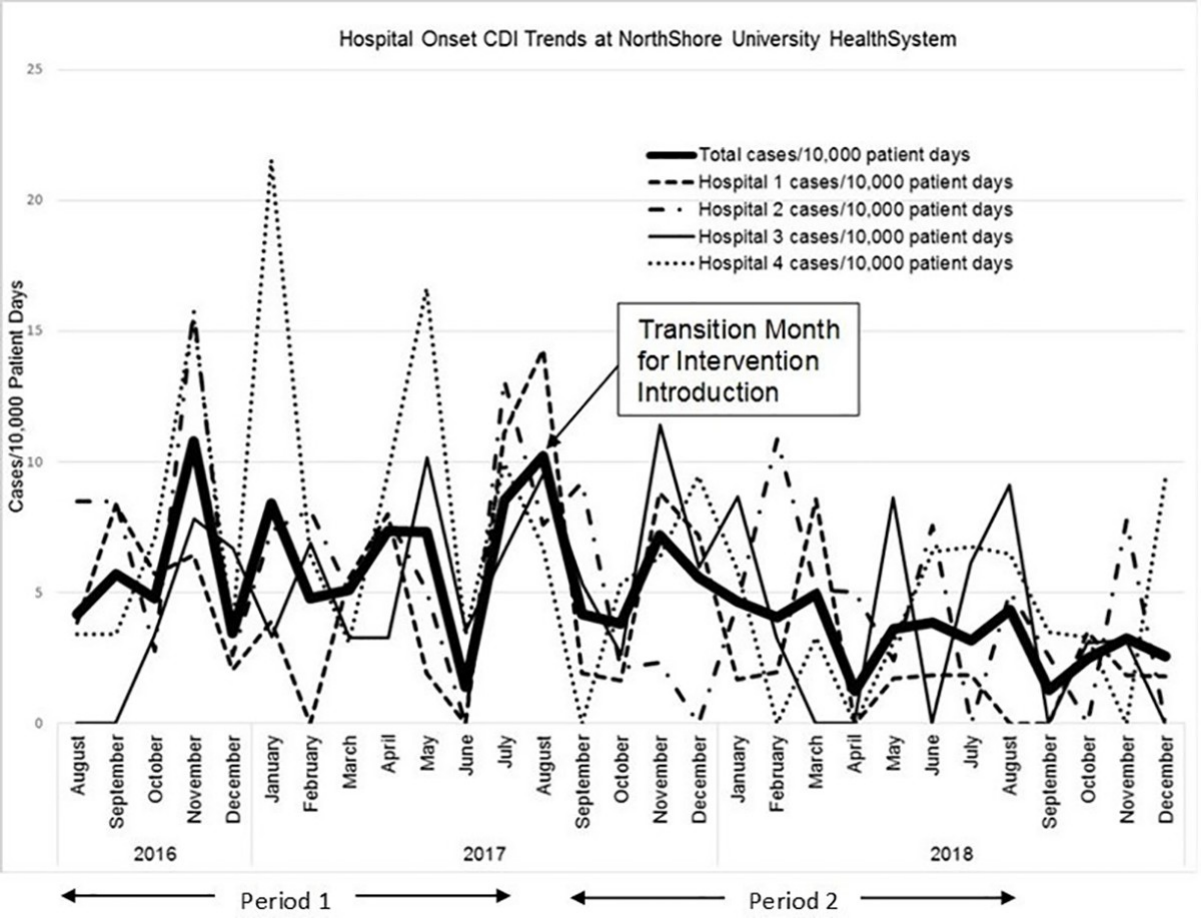
Monthly incidence of HO-CDI during 29 months of observation (S1 Table).

The changes in HO-CDI during the complete 4-hospital targeted admission testing initiative are shown in Fig 2 (S1 Table). We assessed the potential impact of seasonality on our results by comparing the quarterly CDI rates in Periods 1 and 2. For Period 1, the quarterly rates were 4.88, 7.44, 5.73, and 5.8 cases per 10,000 patient days respectively. In Period 2 the quarterly rates were 5.01, 4.78, 3.27, and 3.79 cases per 10,000 patient days. Only the first quarter of Period 1 had a lower rate than any of the quarters in Period 2, which was the first quarter of the intervention year. Over these two yearly periods there was no apparent seasonality with the ranking of disease rate by quarter (highest to lowest) being quarters 2, 4, 3, and 1 in Period 1 versus quarters 1, 2, 4, and 3 in Period 2.

**Fig 3:**
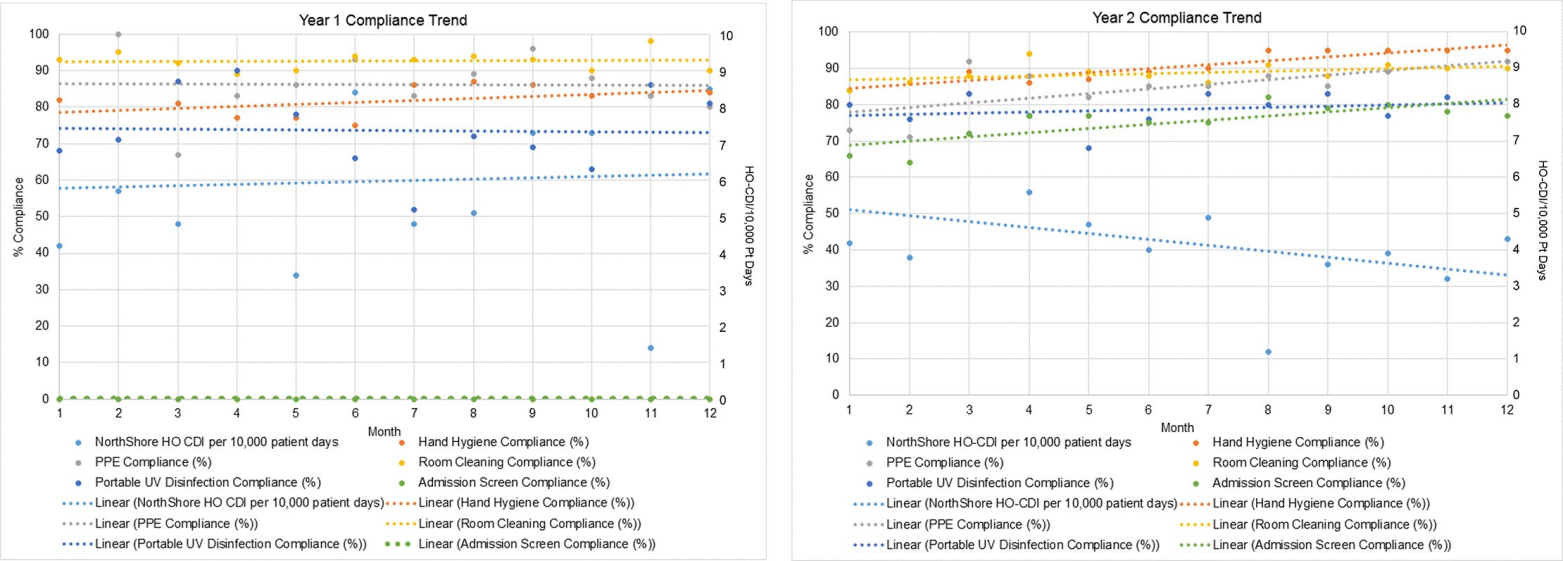
**a.** and **b.** Compliance with infection control practices designed to reduce risk of HO-CDI (3a represents Period 1 and 3b represents Period 2; S2 Table).

The demographics of the NorthShore inpatient population during the time of the admission testing initiative plus the final four months of 2018 are in Table 2. There were no significant differences between this population and that in Period 1. Targeted testing captured 30% of admissions and 8.3% of tested patients were positive. The mean admission testing compliance during Period 2 was 75%, beginning at 66% and ending at 77%. Patients who declined testing were included in the analysis population–there were no exclusions. In the year prior to the intervention (Period 1) there were 63,057 admitted patients and the rate of HO-CDI increased from 5.9 to 6.1 cases/10,000 patient days (Fig 3A; *p*>0.2). Comparison of HO-CDI rates in Periods 1 and 2 showed a significant disease decrease in HO-CDI during Period 2, to 4.23 cases/10,000 patient days (Fig 2; *p* = 0.02). Continuing this admission surveillance initiative for an additional 4 months provided a total of 85,492 admissions. During the final 9 months of 2018 the HO-CDI incidence was 2.9 cases/10,000 patient days (Fig 2; *p*<0.0001 compared to Period 1). In our healthcare system this equals less than 1 case of HO-CDI/1,000 admissions (0.82 cases/1,000 admissions). There were no reported adverse events from admission testing.

**Table 2:** Characteristics of the NorthShore inpatient population during the Infection control intervention initiative.

Characteristic	Number of Patients	Percentage
Gender		
Male	37,088	43.4%
Female	48,404	56.6%
Ethnicity		
Hispanic/Latino	3,962	4.6%
Not Hispanic	81,530	96.4%
Race		
African American	5,629	6.6%
Asian	3.548	4.1%
Caucasian	62,248	72.8%
Other	14,068	16.5%
Insurance		
Private	29,527	34.5%
Medicaid	7,145	8.4%
Medicare	46,040	53.9%
Medicare Advantage	1,427	1.7%
Other	539	0.6%
Uninsured	814	1%
Surgery during admission	30,701	35.9%
ICU during admission	14,564	17%
Mortality during admission	2,206	2.6%
Readmitted within 30 days	1,404	1.6%
Measure	Mean	Standard Deviation
Age	68 years	19 years
Length of hospital stay	4.9 days	5 days
ICU length of stay	4 days	4.7 days
Body Mass Index	28 kg/m^2^	7.1 kg/m^2^

We also measured changes in any of the other ongoing Infection Control practices intended for prevention of CDI between Periods 1 and 2, as shown in Fig 3A and 3B, respectively (S2 Table). Hand hygiene significantly improved in both periods (*p*<0.001) but had no association with the increased HO-CDI rate in Period 1. Compliance with this practice never falling below 78% (*r*(22) = -.50). Room cleaning compliance had no significant change in Period 1 and significantly worsened in Period 2 (*p* = 0.005), indicating no association with lower HO-CDI rates (*r* (22) = -.04). Portable ultraviolet light disinfection compliance significantly decreased in Period 1 and had no change in Period 2, again implying no impact on the reduced HO-CDI after the admission surveillance initiative (*r*(22) = -.09). Use of personal protective equipment (PPE) had significant changes in both Periods 1 and 2, worsening in Period 1 and improving in Period 2 (*r*(22) = .14). However, the overall change was from 87% to 92% compliance across both periods, and the return to the 87% compliance of Period 1 did not occur until 9 months into Period 2, well after the HO-CDI incidence was declining.

The rate of clinical diagnostic testing in Period 2 was 58.05 tests per 1,000 admissions, which was 58.8%% of that in Period 1 (98.7 tests per 1,000 admissions; *p*<0.001). The percentage of clinical tests for CDI that were negative was 85.3% (5,312 tests) in Period 1 and 82.5% (3,004 tests) in Period 2 (*p* = 0.27). While the 41% reduction in testing was greater than the 29% lowering of clinical CDI rates, this was not accompanied by a significant change in the negative testing rate, suggesting that clinical practice impacting when to test had not changed and that the lower amount of testing was due to encountering fewer potential cases of CDI. Interestingly, during the final 9 months of the reporting period (April through December, 2018) there was a 49% reduction in CDI compared to baseline that compares well to the reduction in testing for possible CDI.

**Fig 4:**
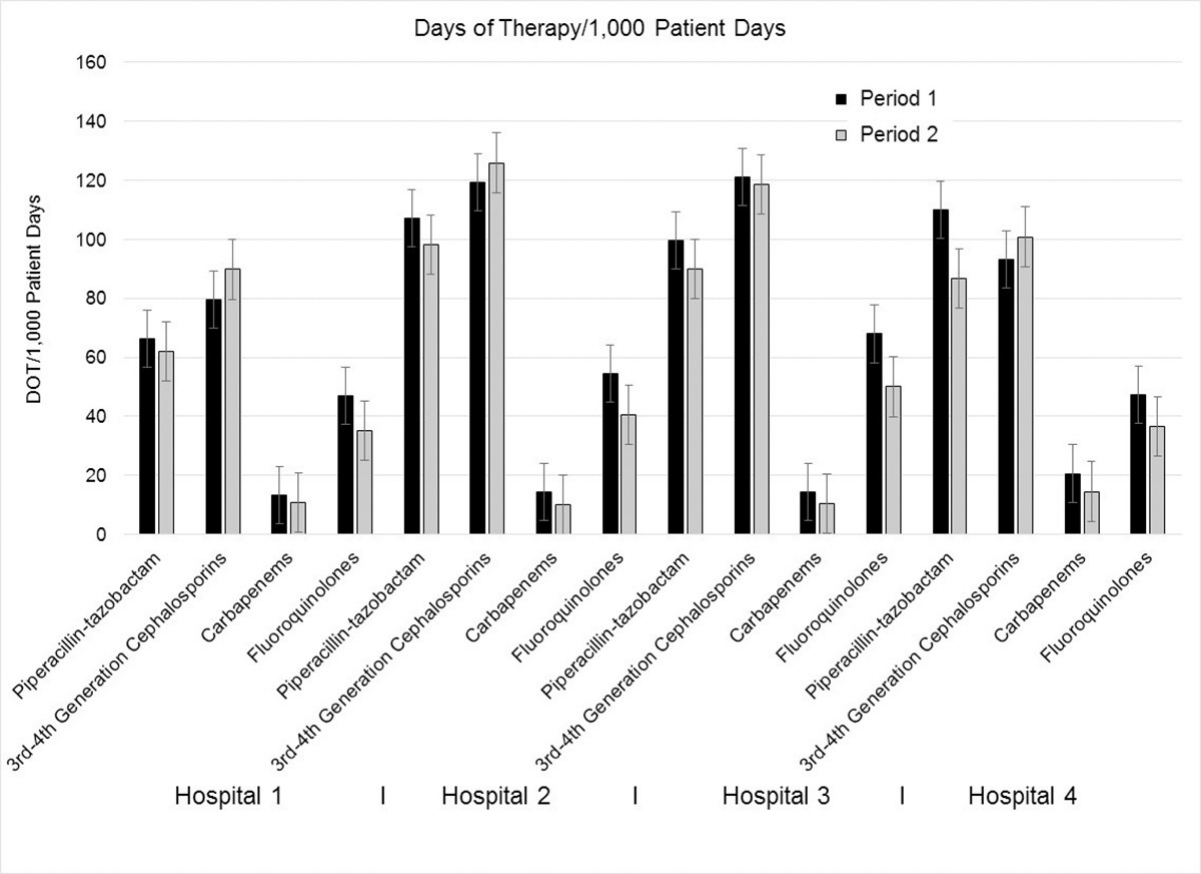
Antimicrobial agent use (in days of therapy per 1,000 patient days) between the two study periods depicted as total use ±1 S.E.; S3 Table).

The antimicrobial use data is shown in Fig 4 (S3 Table), and there was a modest change in prescribing between the two observation periods, with an overall reduction of 8.1% for the monitored antimicrobial agents. The antimicrobials included in Fig 4 represented 75.7% of total inpatient antibacterial agents prescribed during Period 1 and 78.3% in Period 2. Since there was a (non-significant; *p*>0.19) reduction in fluoroquinolone use at all four hospitals, we reviewed the antimicrobials received by all patients with a diagnosis of CDI in Periods 1 and 2 to determine if there was a change in the pattern of fluoroquinolone receipt risk. In Period 1 there were 12 of 105 total cases (11.4%) who received a fluoroquinolone prior to the onset of CDI, and in three of these it was the only agent. For Period 2, 13 of 83 CDI cases (15.7%) were exposed to a fluoroquinolone before clinical CDI, and in four it was the only agent prescribed. Therefore it does not appear that prescribing of fluoroquinolones significantly (*p* = 0.60) impacted those patients developing CDI when comparing the two study periods.

After the first year the initiative was continued and when the trend line for HO-CDI in Period 2 (Fig 2) was extended to the end of December 2018, the change (reduction trend) in HO-CDI was increasingly significant (*p*<0.004). Extending the baseline data back through October 2015 provided a clinical CDI rate before the intervention of 6.96 cases per 1,000 patient days (221 cases over 317,602 patient days) compared to 3.78 cases per 1,000 patient days (98 cases over 259,124 patient days) through the end of 2018 (*p*<0.001).

## Discussion

In this quality improvement, stepped-wedge initiative we demonstrated that targeted admission surveillance for toxigenic *C*. *difficile* colonization, followed by appropriate infection control practice, can significantly reduce the incidence of HO-CDI to a very low level (<1 case/1,000 admissions), even when using NAAT diagnostic testing as the only laboratory test. We completed testing and reporting in under one day from admission. This was done in a setting where multiple other practices appear to have had no impact on reducing hospital onset clinical *C*. *difficile* disease. To our knowledge, this is the first report that has deployed such an Infection Prevention and Control program as routine clinical practice at multiple inpatient facilities.

We performed a literature review using PubMed.gov (MEDLINE) and Google.com with the search terms *Clostridium difficile*, *Clostridioides difficile*, active surveillance testing, and *C*. *difficile* prevention and control from 2005 through June 2019. In the recent literature there have been two reports that colonized persons are a significant component of transmission within the hospital and that admission surveillance for toxigenic *C*. *difficile* may be useful [10,11]. Caroff and coworkers reported that most cases of HO-CDI do not have a genetic link to another patient’s strain and that clinical disease may be due to activation of *C*. *difficile* colonization present-on-admission or a new transmission from an asymptomatic patient [11]. Mawer and colleagues found that patients with stool harboring a toxigenic *C*. *difficile* strain, where the immunoassay stool toxin test was negative, appeared responsible for 25% of transmission events to others when such transmission could be ascertained [12]. Furthermore, they suggested that contact isolation of asymptomatically colonized patients, who are approximately 10-fold more numerous than disease patients, may result in a larger reduction in transmission–and presumably less clinical disease [12]. Thus supporting recommendations for new control measures including admission screening suggested as a possible intervention for lowering the risk of HO-CDI in the current Up-to-Date document on prevention and control of this disease [7].

Earlier, Longtin and colleagues reported the impact of toxigenic *C*. *difficile* admission testing, using a commercial nucleic acid amplification test, on the incidence of CDI in their facility when patients coming through their emergency department were tested [8]. They found a CDI reduction of more than 50% (*p*<0.001), from 0.69/1,000 patient days before the intervention to 0.3 cases/1,000 patient days after screening was implemented [8]. This rate is very similar to the 0.289 cases/1,000 patient days (e.g., 2.89 cases/10,000 patient days) we observed during the final 9 months of 2018. More recently another report appeared indicating the utility of admission screening focused on surgical patients during an outbreak setting, again showing benefit by lowering expected cases [29]. A recent national survey suggested that surveillance for *C*. *difficile* is occurring in 4% of U.S. hospitals, but not in an organized admission surveillance program [30]. This suggests that some screening for colonization is taking place, but the data to inform practitioners as to what might be beneficial is lacking.

The cost effectiveness for implementing admission surveillance testing has been evaluated. Modeling the cost effectiveness found a financial savings for every level of contact precaution intervention when the prevalence of *C*. *difficile* colonization exceeded 5% [31]. With our positive test result rate being 8.3%, the benefit in this model is realized. Their model used a cost of surveillance testing up to $15.88 [32]; if one performs targeted surveillance and realizes the same benefit for disease reduction, then the overall cost of testing can easily achieve this level. One important financial incentive to control the rate of CDI in United States hospitals is the Centers for Medicare and Medicaid Services (CMS) classification of CDI as a hospital acquired condition (HAC) with potential for reduced reimbursement if disease rates are too high [33]. Each year the HAC reduction program saves CMS $350 million by reducing payments to hospitals, thus incentivizing United States healthcare to reduce hospital acquired infections [33]. Combining the impact of this CMS program with surveillance data from the CDC that found the CDI disease rate higher in 2016 than in 2012 and 2013 [34], admission surveillance testing can be a useful intervention to lower HO-CDI cost.

It is somewhat surprising that all the other practices (Table 1) we had previously introduced did not lower the HO-CDI rate. Current guidance and recommendations for prevention and control of CDI have assessed each of these practices [6, 35], with the most current review being that of McDonald and Kutty [6]. Since *C*. *difficile* forms spores, it is accepted that a sporicidal agent, such as bleach, is required for disinfection of clinical areas where patients with CDI have resided. However, the data supporting enhanced room disinfection at the time of discharge is somewhat contradictory. In a large, multicenter, crossover trial involving 21,395 patients, the incidence of infection or colonization with a resistant organism was not reduced when bleach was used to enhance routine room disinfection. In addition, when *C*. *difficile* was the specific target organism, UV addition to bleach room disinfection did not provide any added benefit in the primary analysis [36]. In the secondary analysis of this same data set the authors found that for *C*. *difficile* the hospital-wide risk was reduced when portable UV cleaning was used, but not if bleach or UV plus bleach disinfection was done [37]. Interestingly, the reference group for this study had a 10% higher risk during the UV period compared to the other two disinfection method periods, and the actual rate of *C*. *difficile* disease risk was lower in the bleach or UV plus bleach periods as compared to the UV alone period [37].

Hand hygiene is recommended to be performed using soap and water as opposed to an alcohol-based hand rub, especially during outbreaks, since the spores of *C*. *difficile* are resistant to alcohol [6, 35]. At least one well done study has demonstrated better removal of *C*. *difficile* from hands using soap and water [38]–we followed that practice. A frequently cited statistic is that the baseline rate of healthcare worker compliance with recommended hand hygiene practice is 40%, ranging from 5% to 81% [39]. As can be seen in Fig 3, our performance was consistently in the upper range (≥78%) of reported compliance for both the baseline and intervention initiative periods. In view of all the infection control practices we had deployed prior to initiating admission surveillance for toxigenic *C*. *difficile*, we compared the trends in compliance for each of these with the incidence of HO-CDI and found that only the admission surveillance initiative appeared to have a direct, positive impact on reducing HO-CDI incidence.

Antimicrobial stewardship is also considered a key tool for reducing CDI rates [6, 35]. We did not have a program targeting reduced use of any particular antimicrobial agent class, and as can be seen from Fig 4, the amount of antimicrobial agents prescribed were consistent over both study periods. Piperacillin-tazobactam along with third plus fourth generation cephalosporins were the most heavily used agents, with piperacillin-tazobactam having 11.2% less use and the cephalosporins 6.5% more use in Period 2 compared to Period 1. Kundrapu and colleagues investigated the impact of commonly used antimicrobials (e.g., piperacillin-tazobactam, ampicillin, linezolid, carbapenems, cephalosporins, and ciprofloxacin) on colonization and found that only piperacillin-tazobactam was associated with significantly less *C*. *difficile* in the stool [40]. For our initiative, even with a reduction in piperacillin-tazobactam use in Period 2 we realized a lowering of HO-CDI rates after deployment of the targeted admission surveillance program. From Fig 2 through 4, it appears that the targeted admission surveillance program had the greatest, and perhaps only, impact on lowering HO-CDI to a very low rate of clinical disease.

We also monitor the effectiveness of our Infection Control programs to reduce transmission of other nosocomial pathogens, namely MRSA, vancomycin resistant enterococci (VRE) and Gram negative MDRO pathogens. Throughout the time of this report there was no change in the frequency of hospital-onset infections for any of these pathogens. MRSA was maintained at a rate below 0.3 blood stream infections (BSI) per 10,000 patient days, VRE BSI below 0.2 per 10,000 patient days, and Gram negative MDRO infections within any of our hospital intensive care units below 0.2 infections per 10,000 patient days.

There are limitations to our report. The primary one is that this was not a randomized trial, but rather a stepped-wedge (e.g., before: after) infection control initiative. In support of our findings, we first performed a single-hospital pilot intervention that demonstrated a significant reduction of HO-CDI, which was then followed by the same outcome when the intervention initiative was applied at all 4 NorthShore hospitals. The other major limitation is that there were multiple practices already underway at the time the targeted admission surveillance for toxigenic *C*. *difficile* colonization was begun. We have addressed this concern by evaluating compliance trends for all recommended infection control practices as well as monitoring antimicrobial agent use. It is also possible that physician knowledge their patient was colonized with toxigenic *C*. *difficile* affected subsequent prescribing of antimicrobial agents by the care provider. We were not able to monitor this, but should such a behavior change occur it would be considered a benefit of knowing the patient’s carrier status in an attempt to prevent HO-CDI.

A minor limitation is the cost of the program and potential unnecessary patient isolation. If one develops a targeted admission surveillance program based on risk factors for *C*. *difficile* colonization, which we have done for our system-wide surveillance implementation, one will likely test 1/3 of admissions (our practice) thereby reducing the cost to an effective level. Also, based on the performance of the test we used, the expectation is that there will be one unnecessary isolated patient per 200 admissions [21], which we consider an acceptable number resulting from a false positive test result. Finally, one could argue that our use of a NAAT for routine clinical diagnostic testing could result in an over estimation of the burden of HO-CDI at our organization [41]. However, using a NAAT for routine diagnostic testing throughout this study demonstrated that the incidence of HO-CDI can be reduced to a very low level even when using the most sensitive diagnostic test available to the clinical laboratory.

Useful future research could investigate those prevention strategies that are successful and needed. It is very likely that all the practices contained in Table 1 are not required for a successful program to lower HO-CDI. A systematic approach to evaluate all these potential strategies can be undertaken that reduces the cost for prevention of this healthcare-associated infectious disease.

In conclusion, *C*. *difficile* is a challenging organism that seems to be increasingly adaptive to the hospital environment [42], likely posing an ongoing, still emerging threat to patients. Two recent reports performing *C*. *difficile* surveillance for quantifying risk factors leading to CDI concluded that identification of carriers could lead to reduced spread of this disease and that targeted screening of high risk groups should be considered [43,44]. We have provided evidence demonstrating admission *C*. *difficile* surveillance testing is an important tool for lowering HO-CDI that we accomplished with an incidence of under 1 case of HO-CDI per 1,000 admissions during the final 9 months of 2018. Multiple other practices had been deployed but they were not successful in achieving this goal. We believe this initiative is generalizable to U.S. healthcare since our hospitals range from community facilities to academic teaching hospitals. The admission surveillance program is now part of our routine Infection Control program at NorthShore.

## Supporting information

S1 Checklist TREND statement checklist. (PDF)

S2 Checklist (PDF)

S1 Table Clinical HO-CDI detected (per month) during the time period in the manuscript data (Fig 1). (DOCX)

S2 Table Compliance with Infection control practices (Fig 2). (DOCX)

S3 Table Antimicrobial use in days of therapy/1,000 patient days (Fig 3). (DOCX)

S1 Flow diagram (PDF)

S1 Protocol (PDF)
