# Supplementary material for: Reduced Clostridioides difficile infection in a pragmatic stepped-wedge initiative using admission surveillance to detect colonization
Source: PLoS One. 2020 Mar 19;15(3):e0230475. doi: 10.1371/journal.pone.0230475 (PMC7082001; doi:10.1371/journal.pone.0230475)
Supplement: S2 Checklist — (PDF) [file pone.0230475.s002.pdf]

NorthShore University HealthSystem

NorthShore University Health System Research Institute
Quality Improvement (QI) Checklist

Investigators are encouraged to use the “QI Checklist” to help determine whether the proposed activity is considered a Quality Improvement project or whether IRB review is required. (Also see Clinical Research Checklist)

1. Purpose   Yes ☒   No ☐
   a. Is the project intended to improve the process/delivery of care while decreasing inefficiencies? or
   b. Is the project intended to describe lessons learned? or
   c. Is the project intended to produce a new strategy or intervention?

2. Project Staff   Yes ☒   No ☐
   a. Is the proposed project conducted by the clinicians and/or staff who provide care or who are responsible for the performance quality in the work area?

3. Project Design   Yes ☒   No ☐
   a. Is the project designed with the intent to implement improvement for the benefit of the hospital? Or
   b. Is the project design flexible, including rapid and incremental changes such as when new facts are discovered?

4. Recruitment   Yes ☒   No ☐
   a. Will the project involve a sample of the population (staff or patients) ordinarily seen in the work setting where the project will take place?

5. Consent   Yes ☒   No ☐
   a. Will the planned activity only require consent that is normally sought in clinical practice and could the activity be considered part of the usual care?

6. Benefits   Yes ☒   No ☐
   a. Is it true that most of the patients where the planned activity will take place could potentially benefit now or in the future from the project?

7. Risk   Yes ☒   No ☐
   a. Is the risk to the participants no greater than what is involved in the care they are already receiving? or
   b. Can the burden of participating in the activity be considered acceptable or ordinarily expected when reforms are being introduced to the way care is provided?

If the answers to **ALL** of these questions is **Yes**, then the activity is a QI project and does not involve human subject research. IRB review is not required.
If the answer to **ANY** of these questions is **NO**, please consult with the IRB. IRB review may be required.
