# Supplementary material for: Reduced Clostridioides difficile infection in a pragmatic stepped-wedge initiative using admission surveillance to detect colonization
Source: PLoS One. 2020 Mar 19;15(3):e0230475. doi: 10.1371/journal.pone.0230475 (PMC7082001; doi:10.1371/journal.pone.0230475)
Supplement: S1 Table — (DOCX) [file pone.0230475.s003.docx]

S**upplemental Table 1. Clinical HO-CDI detected (per month) during the time period in the manuscript data (Fig 1).**

|  |  | Total cases/10,000 patient days | Hospital 1 cases/10,000 patient days | Hospital 2 cases/10,000 patient days | Hospital 3 cases/10,000 patient days | Hospital 4 cases/10,000 patient days |
| --- | --- | --- | --- | --- | --- | --- |
| 2016 | August | 4.18 | 3.86 | 8.48 | 0 | 3.39 |
|  | September | 5.72 | 8.35 | 8.48 | 0 | 3.39 |
|  | October | 4.76 | 5.69 | 2.78 | 3.34 | 7.08 |
|  | November | 10.8 | 6.43 | 15.81 | 7.84 | 15.5 |
|  | December | 3.44 | 2.05 | 2.59 | 6.7 | 3.54 |
| 2017 | January | 8.4 | 3.9 | 7.35 | 3.31 | 21.56 |
|  | February | 4.78 | 0 | 8.15 | 6.91 | 6.43 |
|  | March | 5.09 | 5.53 | 5.02 | 3.27 | 3.08 |
|  | April | 7.34 | 8.01 | 7.92 | 3.25 | 9.55 |
|  | May | 7.3 | 1.93 | 5.08 | 10.14 | 16.62 |
|  | June | 1.38 | 0 | 0 | 3.56 | 3.35 |
|  | July | 8.52 | 11.1 | 13.11 | 6.61 | 9.93 |
|  | August | 10.22 | 14.31 | 7.6 | 9.55 | 6.7 |
|  | September | 4.16 | 1.91 | 9.23 | 5.34 | 0.00 |
|  | October | 3.80 | 1.65 | 2.10 | 2.61 | 5.34 |
|  | November | 7.21 | 8.81 | 2.31 | 11.43 | 6.35 |
|  | December | 5.59 | 7.19 | 0.00 | 5.96 | 9.44 |
| 2018 | January | 4.66 | 1.68 | 4.59 | 8.66 | 5.86 |
|  | February | 4.04 | 1.96 | 10.87 | 3.27 | 0.00 |
|  | March | 4.94 | 8.59 | 5.11 | 0.00 | 3.29 |
|  | April | 1.25 | 0.00 | 5.03 | 0.00 | 0.00 |
|  | May | 3.59 | 1.70 | 2.40 | 8.62 | 3.14 |
|  | June | 3.85 | 1.83 | 7.54 | 0.00 | 6.54 |
|  | July | 3.17 | 1.82 | 0.00 | 6.14 | 6.76 |
|  | August | 4.34 | 0.00 | 5.01 | 9.10 | 6.47 |
|  | September | 1.29 | 0.00 | 2.57 | 0.00 | 3.51 |
|  | October | 2.50 | 3.50 | 0.00 | 3.05 | 3.27 |
|  | November | 3.23 | 1.84 | 7.74 | 3.18 | 0.00 |
|  | December | 2.56 | 1.81 | 0.00 | 0.00 | 9.31 |
