# Supplementary material for: Reduced Clostridioides difficile infection in a pragmatic stepped-wedge initiative using admission surveillance to detect colonization
Source: PLoS One. 2020 Mar 19;15(3):e0230475. doi: 10.1371/journal.pone.0230475 (PMC7082001; doi:10.1371/journal.pone.0230475)
Supplement: S2 Table — (DOCX) [file pone.0230475.s004.docx]

S**upplemental Table 2. Compliance with Infection Control practices (Fig 2).**

| Date | | Month | | NorthShore HO CDI per 10,000 patient days | | Hand Hygiene Compliance (%) | | PPE Compliance (%) | | Room Cleaning Compliance (%) | | Portable UV Disinfection Compliance (%) | | Admission Screen Compliance (%) | |
| --- | --- | --- | --- | --- | --- | --- | --- | --- | --- | --- | --- | --- | --- | --- | --- |
| 2016 | | August | | 42 | | 82 | | 91 | | 93 | | 68 | | 0 | |
|  | | September | | 57 | | 80 | | 100 | | 95 | | 71 | | 0 | |
|  | | October | | 48 | | 81 | | 67 | | 92 | | 87 | | 0 | |
|  | | November | | 108 | | 77 | | 83 | | 89 | | 90 | | 0 | |
|  | | December | | 34 | | 77 | | 86 | | 90 | | 78 | | 0 | |
| 2017 | | January | | 84 | | 75 | | 93 | | 94 | | 66 | | 0 | |
|  | | February | | 48 | | 86 | | 83 | | 93 | | 52 | | 0 | |
|  | | March | | 51 | | 87 | | 89 | | 94 | | 72 | | 0 | |
|  | | April | | 73 | | 86 | | 96 | | 93 | | 69 | | 0 | |
|  | | May | | 73 | | 83 | | 88 | | 90 | | 63 | | 0 | |
|  | | June | | 14 | | 83 | | 83 | | 98 | | 86 | | 0 | |
|  | | July | | 85 | | 84 | | 80 | | 90 | | 81 | | 0 | |
| September | | 42 | | 84 | | 73 | | 84 | | 80 | | 66 | |  |  |
| October | | 38 | | 86 | | 71 | | 86 | | 76 | | 64 | |  |  |
| November | | 72 | | 89 | | 92 | | 88 | | 83 | | 72 | |  |  |
| December | | 56 | | 86 | | 88 | | 94 | | 77 | | 77 | |  |  |
| 2018 January | | 47 | | 87 | | 82 | | 89 | | 68 | | 77 | |  |  |
| February | | 40 | | 89 | | 85 | | 88 | | 76 | | 75 | |  |  |
| March | | 49 | | 90 | | 85 | | 86 | | 83 | | 75 | |  |  |
| April | | 12 | | 95 | | 88 | | 91 | | 80 | | 82 | |  |  |
| May | | 36 | | 95 | | 85 | | 88 | | 83 | | 79 | |  |  |
| June | | 39 | | 95 | | 89 | | 91 | | 77 | | 80 | |  |  |
| July | | 32 | | 95 | | 90 | | 90 | | 82 | | 78 | |  |  |
| August | | 43 | | 95 | | 92 | | 90 | | 80 | | 77 | |  |  |
