# Supplementary material for: Reduced Clostridioides difficile infection in a pragmatic stepped-wedge initiative using admission surveillance to detect colonization
Source: PLoS One. 2020 Mar 19;15(3):e0230475. doi: 10.1371/journal.pone.0230475 (PMC7082001; doi:10.1371/journal.pone.0230475)
Supplement: S3 Table — (DOCX) [file pone.0230475.s005.docx]

S**upplemental Table 3. Antimicrobial use in days of therapy/1,000 Patient Days (Fig 3).**

|  |  | **Hospital 1 Antimicrobial use** | | |
| --- | --- | --- | --- | --- |
|  | Piperacillin-tazobactam | 3rd-4th Generation Cephalosporins | Carbapenems | Fluoroquinolones |
| Period 1 | 66.2 | 79.6 | 13.3 | 47 |
| Period 2 | 62 | 89.8 | 10.8 | 35.1 |

|  |  | **Hospital 2 Antimicrobial use** | | |
| --- | --- | --- | --- | --- |
|  | Piperacillin-tazobactam | 3rd-4th Generation Cephalosporins | Carbapenems | Fluoroquinolones |
| Period 1 | 107 | 119.2 | 14.3 | 54.4 |
| Period 2 | 98.1 | 125.9 | 9.9 | 40.4 |

|  |  | **Hospital 3 Antimicrobial use** | | |
| --- | --- | --- | --- | --- |
|  | Piperacillin-tazobactam | 3rd-4th Generation Cephalosporins | Carbapenems | Fluoroquinolones |
| Period 1 | 99.6 | 121 | 14.3 | 67.9 |
| Period 2 | 90 | 118.6 | 10.3 | 50 |

|  |  | | **Hospital 4 Antimicrobial use** | | | | | |  |
| --- | --- | --- | --- | --- | --- | --- | --- | --- | --- |
|  | Piperacillin-tazobactam | | 3rd-4th Generation Cephalosporins | | Carbapenems | | Fluoroquinolones | |  |
| Period 1 | | 109.9 | | 93.2 | | 20.6 | | 47.4 | |
| Period 2 | | 86.8 | | 100.8 | | 14.5 | | 36.6 | |
