# Supplementary figures and images for: Reduced Clostridioides difficile infection in a pragmatic stepped-wedge initiative using admission surveillance to detect colonization
Source: PLoS One. 2020 Mar 19;15(3):e0230475. doi: 10.1371/journal.pone.0230475 (PMC7082001; doi:10.1371/journal.pone.0230475)

## CONSORT 2010 Flow Diagram

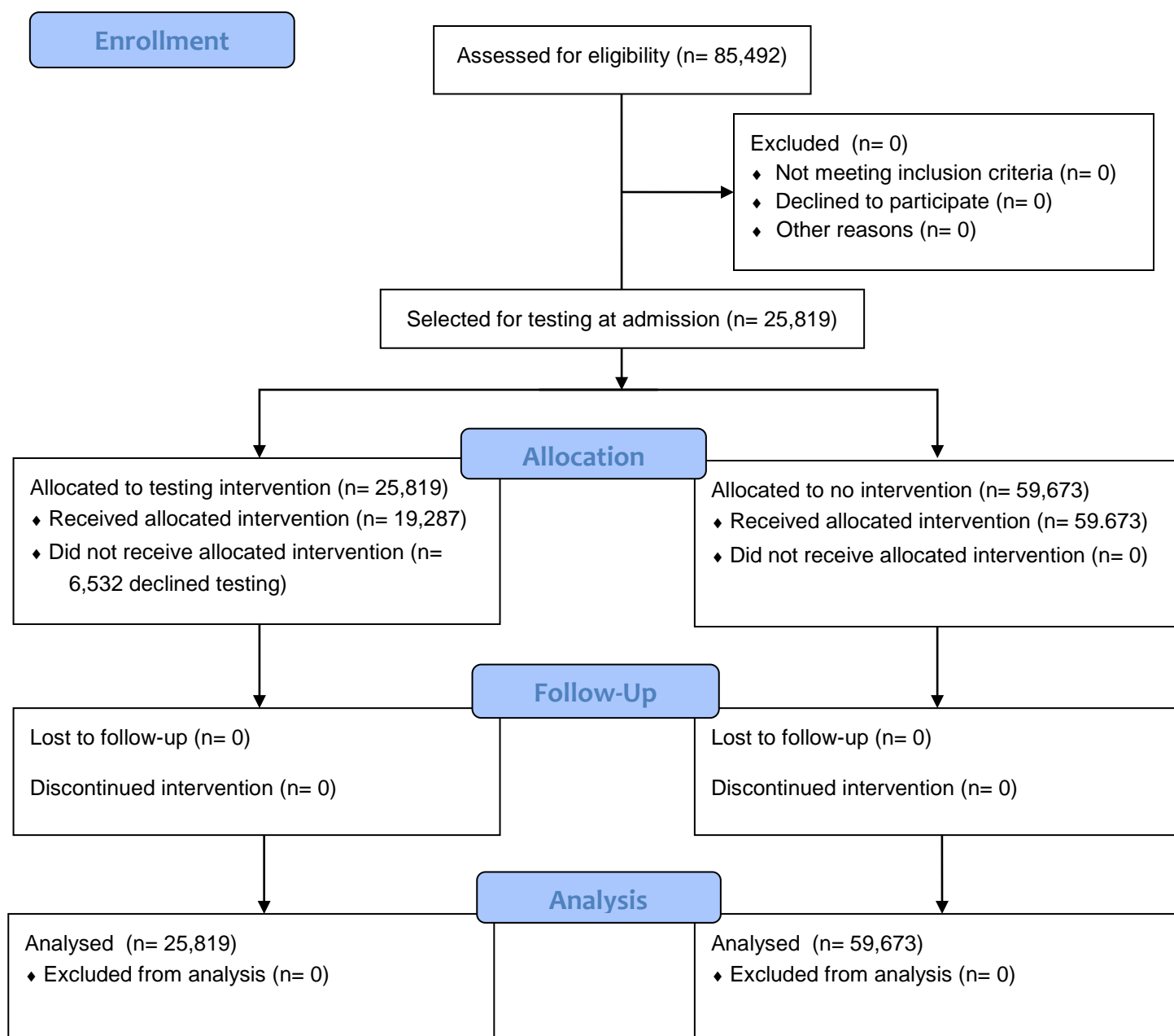

Supplement: S1 Flow diagram — (PDF) [file pone.0230475.s006.pdf]
