# Supplementary material for: Reduced Clostridioides difficile infection in a pragmatic stepped-wedge initiative using admission surveillance to detect colonization
Source: PLoS One. 2020 Mar 19;15(3):e0230475. doi: 10.1371/journal.pone.0230475 (PMC7082001; doi:10.1371/journal.pone.0230475)

Study Protocol for All Admission Surveillance (6/8/2016)

Background

Clostridium difficile infection (CDI) remains a constant, even increasing, clinical infection threat in the United States and Europe. New preventive strategies are urgently needed. Current control measures do not target asymptomatic carriers, despite evidence that they can contaminate the hospital environment and health care workers' hands; a risk to potentially transmit *C. difficile* to other patients. Of special importance is that not only is disease prevalence increasing, but severity of illness and mortality also is on the rise. We have implemented many intervention practices at our healthcare system including bleach cleaning of rooms with CDI patients, monitoring of environmental services' room cleaning, hand hygiene education and even UV light disinfection of rooms harboring CDI patients. To date, none have been consistently effective. We believe that CDI is much like MRSA, in that many more patients are colonized than infected, and one (perhaps the optimal) way to curtail disease is to interrupt transmission by identifying carriers and placing them into contact (glove and gown) precautions.

We propose to test this hypothesis with a quality improvement program initiative at NorthShore. Our primary goal is to demonstrate that surveillance testing for *C. difficile* colonization at the time of admission and contact precaution isolation of those positive will reduce spread of the organism as well as lower clinical CDI. Our secondary goal is demonstrate that such a program is cost effective.

The primary outcome measure is reduced CDI disease. The secondary outcome measures are reduced cost for the care of patients with CDI (by avoiding disease) and meeting CMS threshold criteria for CDI disease rates in our acue care facilities.

Study Methods

We have collected CDI disease rates for 10 years. One of our hospitals admits a high number of patients from long term care facilities (LTCFs) and currently has a hospital-onset (HO) CDI rate of 1.1 cases/1,000 patient days. The figure below presents their CDI data for calendar 2015, and the nosocomial CDI rate at this facility has been rising during the first quarter of 2016. The hospital admits 25 patients per day, which translates to 2,250 admission tests in 3 months, and for this facility represents approximately 11,000 patient days. We plan to introduce peri-rectal (intra-anal) swab testing for *C. difficile* on all admissions for 3 months, accompanied by isoation of positive patients, which we hypothecize will lower the rate to 0.3 cases/1,000 patient days. In absolute numbers, the goal is to reduce the rate from 4 nosocomial cases per month down to 1 nosocomial case per month. This will provide a sample size that can demonstrate a level of sigificance at $p<.05$ for disease reduction with the intervention when compared to the time frame prior to the admission testing intervention. The testing period can be extended if we have not reached the estimated number of consenting patients within the first 3 months of the planned program.

Those patients positive will be placed into contact precaution isolation (gown and gloves worn by all healthcare workers). We will be collecting a double headed swab (Culturette) on all patients, so those testing positive will also have the second swab cultured for *C. difficile* that will be archived for further testing. We will also validate the procedure by culturing a similar number of patients who have a negative Roche C. Diff Cobas test (the first negative patient tested after each positive *C. difficile* result).

As a supplemental data set, our system implemented all hospital inpatient point prevalence surveys (rectal swabs) for CDI and MDRO Gram negative bacteria in the month of June 2016.

If we assume that this proposed program can begin shortly after the new point prevalence NorthShore program, we will have before and after point prevalence data to demonstrate reduction in transmission. In the past we have found the point prevalence of *C. difficile* at the target hospital to be 12% of inpatients. Thus, for example, if the admission prevalence is 5% this would demonstrate that there is considerable spread of *C. difficile* in the hospital after admission. If the proposed admission screening program is successful, we might expect the second point prevalence (planned for September) to be reduced to the same rate as the admission prevalence because we have prevented spread. If this is found, we will have demonstrated that both transmission and disease are reduced by implementing the admission screening program. This limitation of this supplemental analysis is that the number of patients sampled and the rate of positive results may be too small to reach a valid threshold for analysis.

Statistical significance will be determined using the Chi Square/Fisher exact test, with $p \leq 0.05$ being required to determine significance.

Study Period

The planned study period is 3 months - an extension for an additional 6 months can occur based on the initial 3 month data analysis. All admissions to the target hospital will be tested, and the program will continue until at least 2,000 patients have taken part. There are no exclusion criteria. The Department of Nursing has agreed that admitting nurses or patient care technicians (PCTs) will collect the perirectal swab sample specimen.

Testing Plan

Patients admitted to the target hospital will have a peri-rectal swab collected at the time of admission. This will be tested by the Roche cobas C. Diff assay. Testing will be done 6 days per week at the central hospital (Evanston Hospital). We also anticipate that we might be able to develop an algorithm to detect patients at risk for harboring *C. diffiicle* at the time of admission from the data collected in the pilot program. If this is true, then we will be able to test only targeted admissions should the program be deployed to all NorthShore hospitals, as is currently being done for our MRSA admission surveillance program.

Potential Benefit

If this pilot program is successful as we expect, it will be implemented at all 4 of the NorthShore hospitals. A very recent report from Canada (using the BD Cdiff assay) indicates the concept should work (reference 10 below) – we believe our outcome will be even better than the result of this new publication since we will screen everyone - not just ED admissions, and isolation will include gowns as well as gloves. We will be the first US study to show all admission testing works, and as part of our trial we will validate that the cobas assay can be used for a program considering admission surveillance for CDI control. The main objective is patient benefit seen as a significant reduction in healthcare-onset *C. difficile* infection (HO-CDI) that will lower the risk of infection after hospitalization and save patient lives.

References

1. Dallal RM, Harbrecht BG, Boujoukas AJ, et al. Fulminant *Clostridium difficile*: an underappreciated and increasing cause of death and complications. Ann Surg 2002;235:363-72.
2. Dubberke ER, Reske KA, Olsen MA, McDonald LC, Fraser VJ. Short- and long-term attributable costs of *Clostridium difficile*-associated disease in nonsurgical inpatients. Clin Infect Dis 2008;46:497-504.
3. Kuijper EJ, Coignard B, Tull P. Emergence of *Clostridium difficile*-associated disease in North America and Europe. Clin Microbiol Infect 2006;12 Suppl 6:2-18.

4. Loo VG, Poirier L, Miller MA, et al. A predominantly clonal multi-institutional outbreak of *Clostridium difficile*-associated diarrhea with high morbidity and mortality. N Engl J Med 2005;353:2442-9.
5. McDonald LC, Owings M, Jernigan DB. *Clostridium difficile* infection in patients discharged from US short-stay hospitals, 1996-2003. Emerg Infect Dis 2006;12:409-15.
6. Musher DM, Aslam S, Logan N, et al. Relatively poor outcome after treatment of *Clostridium difficile* colitis with metronidazole. Clin Infect Dis 2005;40:1586-90.
7. Pepin J, Valiquette L, Alary ME, et al. *Clostridium difficile*-associated diarrhea in a region of Quebec from 1991 to 2003: a changing pattern of disease severity. CMAJ 2004;171:466-72.
8. Zacharioudakis IM, Zervou FN, Pliakos EE, Ziakas PD, Mylonakis E. Colonization with toxinogenic *C. difficile* upon hospital admission, and risk of infection: a systematic review and meta-analysis. Am J Gastroenterol 2015 Mar;110(3):381-90; quiz 391. doi: 10.1038/ajg.2015.22. Epub 2015 Mar 3.
9. Curry SR, Muto CA, Schlackman JL, Pasculle AW, Shutt KA, Marsh JW, Harrison LH. Use of multilocus variable number of tandem repeats analysis genotyping to determine the role of asymptomatic carriers in *Clostridium difficile* transmission. Clin Infect Dis 2013 Oct;57(8):1094-102. doi: 10.1093/cid/cit475. Epub 2013 Jul 23.
10. Longtin Y, Paquet-Bolduc B, Gilca R, Garenc C, Fortin E, Longtin J, Trottier S, Gervais P, Roussy JF, Lévesque S, Ben-David D, Cloutier I, Loo VG. Effect of detecting and isolating *Clostridium difficile* carriers at hospital admission on the incidence of *C. difficile* infections: A quasi-experimental controlled study. JAMA Intern Med 2016 Apr 25. doi: 10.1001/jamainternmed.2016.0177. [Epub ahead of print]

Figure. Nosocomial CDI cases for the target hospital during calendar 2015.

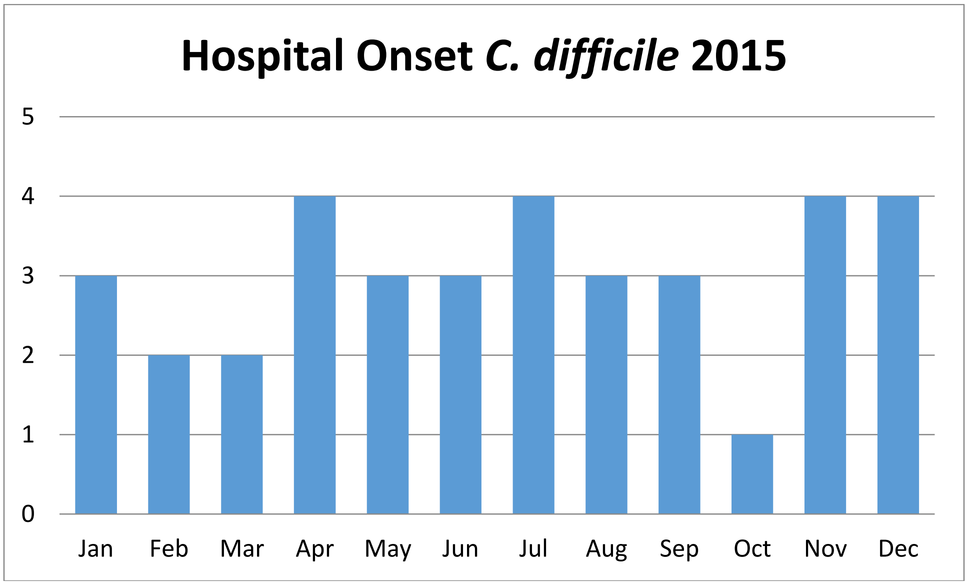

Supplement: S1 Protocol — (PDF) [file pone.0230475.s007.pdf]
